# Evaluation of Different Thermoanalytical Methods for the Analysis of the Stability of Naproxen-Loaded Amorphous Solid Dispersions

**DOI:** 10.3390/pharmaceutics14112508

**Published:** 2022-11-18

**Authors:** Edina Szabó, Anna Haraszti, Petra Záhonyi, Dániel Vadas, István Csontos, Zsombor Kristóf Nagy, Guy Van den Mooter, György Marosi

**Affiliations:** 1 Department of Organic Chemistry and Technology, Budapest University of Technology and Economics (BME), Műegyetem rkp. 3, H-1111 Budapest, Hungary; 2 Department of Pharmaceutical and Pharmacological Sciences, Drug Delivery and Disposition, KU Leuven, Campus Gasthuisberg ON2, Herestraat 49 b921, 3000 Leuven, Belgium

**Keywords:** thermoanalytical methods, glass transition temperature, amorphous solid dispersion, stability, dissolution

## Abstract

The aim of this research was to investigate three thermoanalytical techniques from the glass transition temperature (T_g_) determination point of view. In addition, the examination of the correlation between the measured T_g_ values and the stability of the amorphous solid dispersions (ASDs) was also an important part of the work. The results showed that a similar tendency of the T_g_ can be observed in the case of the applied methods. However, T_g_ values measured by thermally stimulated depolarization currents showed higher deviation from the theoretical calculations than the values measured by modulated differential scanning calorimetry, referring better to the drug-polymer interactions. Indeed, the investigations after the stress stability tests revealed that micro-thermal analysis can indicate the most sensitive changes in the T_g_ values, better indicating the instability of the samples. In addition to confirming that the active pharmaceutical ingredient content is a crucial factor in the stability of ASDs containing naproxen and poly(vinylpyrrolidone-co-vinyl acetate), it is worthwhile applying orthogonal techniques to better understand the behavior of ASDs. The development of stable ASDs can be facilitated via mapping the molecular mobilities with suitable thermoanalytical methods.

## 1. Introduction

The increasing number of newly discovered poorly water-soluble drug candidates encourages the pharmaceutical actors to develop the current formulation strategies and opens up novel methods [1,2]. Otherwise, the properties of the widely used excipients and technologies may create a barrier to the commercialization of effective active pharmaceutical ingredients (APIs) if the final dosage form prepared that way does not fulfill the necessary bioavailability requirements [3]. Amorphous forms can be a possible solution to tackle these challenges since the disordered structure contributes to the enhanced dissolution of poorly water-soluble APIs [4]. However, the majority of the API are thermodynamically stable rather than in the crystalline form. It means that their amorphous status is unstable and can be characterized by higher free Gibbs energy, which pushes the system toward relaxation and eventually crystallization [5,6,7]. If the physical stability of amorphous API decreases, it loses its advantages from the dissolution point of view. Therefore, the authorization bodies strictly require ensuring the stability of the amorphous form at least until the end of the expiration date [8].

As the amorphization of the pure APIs usually results in thermodynamically very unstable systems, polymers are commonly used to increase the stability [9,10]. In this case, the API is distributed in the polymer matrix inhibiting the formation of a crystalline lattice. The thus-formed amorphous solid dispersions (ASDs) are already suitable for pharmaceutical applications since their physical stability is usually much higher than the pure amorphous API. Thanks to this improvement, nearly 30 ASD-containing medicines were approved by the FDA, which indicates the relevance of this formulation strategy in the pharmaceutical field [11]. However, several factors, such as the properties of the polymer, the interactions between the API and the polymer, the preparation method, and the storage condition, can influence the stability of ASDs [12,13,14,15,16,17]. Thus, conscious designing of them and analyzing their amorphous form—usually by X-Ray diffraction (XRD) and differential scanning calorimetry (DSC)—is essential during both development and manufacturing [18].

Prediction of the physical stability of ASDs can facilitate research and development and might accelerate the commercialization process of ASD-loaded pharmaceutical products [19,20]. Therefore, investigation of the molecular properties related to stability has been interesting since the appearance of amorphous pharmaceutical systems and is still a hot topic in this field. Among these features, the glass transition temperature (T_g_) is considered to be one of the key characteristics as it often could foresee instability [21]. Above the T_g_ the molecular mobility is high, the free volume is increasing, and translational movements are starting; therefore, phase separation and crystallization might occur in the ASD of the given API and polymer [22]. In contrast, below the T_g,_ the molecular mobility is decreased, and the system becomes rigid thus, an ideal homogenous ASD could remain in a stable state. Consequently, it is worth storing ASD-containing medicines below the T_g_ of the amorphous form; usually T_g_-50 °C storage temperature is recommended [18,23]. However, it was found recently that the sub-T_g_ transitions also affect the stability of amorphous systems. A more clear correlation was observed in some cases than between the T_g_ and the stability [24,25]. The main difficulty relating to these sub-T_g_ transitions is that their detection can be much harder than the measurement of the T_g_ due to the very small intensity mobilities.

Another benefit of examining the glass transition in the spirit of stability prediction is that several analytical methods are available for measuring T_g_ and investigating the whole relaxation process around the T_g_ [26]. All these techniques detect and record changes in ASDs according to given principles in the function of temperature. The most common method used for T_g_ determination is the DSC, which indicates the glass transition with a ramp in the heat capacity [27]. However, conventional DSC can show overlapping thermal events that complicate the interpretation of the thermograms. For instance, the signal related to T_g_ is hard to separate from enthalpy relaxation or desolvation in the case of polymeric ASDs [26]. A response to the limitation of the conventional DSC was the development of modulated DSC (MDSC) [28,29]. Complementation of the linear heating with a temperature modulation section enabled to distinguish the reversible events (such as glass transition) from non-reversible events (such as the above-mentioned enthalpy recovery or desolvation) [30]. Although the increased sensitivity made MDSC the most popular thermoanalytical tool for examining T_g_ in pharmaceutical amorphous solids, other techniques are also spread in this field [31]. Such are, for example, the different dynamic electrochemical techniques, including dynamic mechanical analysis (DMA) [32], thermally stimulated depolarization current (TSDC) analysis [33], and dielectric relaxation spectroscopy (DRS) [34]. The last one is usually used for the investigation of molecular dynamics around T_g_, while DMA and TSDC methods besides T_g_ measuring are advantageous for examining such amorphous phase separations, which size is under the detection limit of DSC techniques [35,36,37]. To determine these nano and micro inhomogeneities in ASDs, nano- or micro-thermal analysis (µTA) could even be better [38,39]. These methods combine the very high resolution of atomic force microscopy with an extremely small thermal probe, thus providing information about the exact location of the API and polymer phases on nano and microscopic scales within a very short time frame. Overall, it can be stated about the methods suitable for T_g_ determination that as their operation principles differ, the sensitivity and resolution are also distinct. For this reason, the joint application of more analytical techniques can contribute to an in-depth understanding of complex molecular processes in ASDs [40,41]. The better the behavior of the ASDs under development is known, the better the physical stability of the amorphous phase can be estimated.

Related to the comprehensive study of pharmaceutical amorphous solids, the aim of this research was to evaluate MDSC, a TSDC method, and µTA from the T_g_ determination point of view. The investigated model systems contained naproxen (NAP) as API and poly(vinylpyrrolidone-*co*-vinyl acetate) (PVPVA64) as a polymer in different *w*/*w* ratios. One of the reasons for this material selection was the poor glass-forming ability of the API (low T_g_ of amorphous NAP) [42], which can shift the T_g_ of ASDs to lower temperatures as it plasticizes the polymer [43]. This way, the differences between the T_g_ values of the examined compositions with distinct API content can be clear. On the other hand, NAP has a hydrogen bond donor hydroxyl group that enables the formation of intermolecular hydrogen bonds with the polymer which may result in improved physical stability [44]. Consequently, a complex API-polymer ASD system can be examined through the application of NAP and PVPVA64, making the widespread investigation of the selected thermoanalytical methods possible.

Furthermore, the investigation of the relationship between the measured T_g_ values and the stability was also an important aim of the work. Based on the best knowledge of the authors, the three applied thermoanalytical methods were not yet evaluated next to each other in this context before. Consequently, this research might give a novel perspective in the field of the stability investigation of ASDs. According to the results, the highest API content composition had the lowest stability, which was well correlated with the T_g_ values obtained by all three methods. In addition, the order observed by the dissolution tests was similar to the order of the measured T_g_ values. However, the changes of T_g_ after the stress stability test showed differences in the case of the different methods, which suggested that each technique can explain the molecular changes differently. All these results confirm that combining analytical methods relying on different physical principles can help to find compositions expected to be stable. Nevertheless, the prediction of physical stability by using a small amount of sample accelerates the early stage of development. Therefore, accounting for the relevant thermoanalytical techniques in the case of ASDs can be especially significant for the pharmaceutical industry.

## 2. Materials and Methods

### 2.1. Materials

Naproxen (NAP) was purchased from J&K Scientific Ltd. (Lommel, Belgium). PVPVA64 (Kollidon^®^ VA64) was obtained from BASF (Ludwigshafen, Germany). Absolute ethanol (EtOH), dichloromethane (DCM), and 37 *w*/*w*% HCl were purchased from Merck Ltd. (Budapest, Hungary).

### 2.2. Preparation of Amorphous Solid Dispersions

The amorphous solid dispersions of NAP and PVPVA64 were prepared by single-needle electrospinning (SNES). A nozzle with an inner diameter of 0.5 mm was applied for the fiber formation. An NT-35 high voltage direct current supply (MA2000; Unitronik Ltd., Nagykanizsa, Hungary) was connected to the spinneret while a 20 kV electrical potential was set during the experiments. Opposite the needle, a grounded aluminum collector covered with aluminum foil was placed 15 cm far from the spinneret. The solutions containing the API and the polymer were fed by a SEP-10S Plus type syringe pump (Aitecs, Vilnius, Lithuania) with a 10 mL/h dosing rate. The preparation of electrospun amorphous solid dispersions was accomplished at room temperature (25 °C) and at 45 ± 5% relative humidity.

### 2.3. Scanning Electron Microscopy (SEM)

A JEOL JSM 6380LA type scanning electron microscope (JEOL, Tokyo, Japan) was applied to investigate the morphology and size of the electrospun amorphous solid dispersions. A conductive double-sided carbon adhesive tape was used for fixing the specimens to the sample holders. Before the SEM examinations, a thin layer of conducting gold was sputtered to the surface of the samples by a JEOL 1200-type equipment (JEOL, Tokyo, Japan) to avoid electrostatic charging. During the SEM measurements, the accelerating voltage was adjusted to 10 kV, the applied working distance was between 7 and 15 mm, and the spot size was 40 nm. The quantitative evaluation of the photos was performed with a randomized diameter determination method [45].

### 2.4. X-ray Powder Diffraction (XRPD)

A PANalytical X’pert Pro MDP X-ray diffractometer (Almelo, The Netherlands) equipped with a Cu-Kα source and Ni filter was used to investigate the crystalline traces of the electrospun materials. The applied voltage and current were 40 kV and 30 mA, respectively. The measurements were performed in continuous scan mode in a scan range between 2θ angles of 4 and 44° with a step size of 0.0167° and 15 s counting time.

### 2.5. Modulated Differential Scanning Calorimetry (MDSC)

A DSC3+ device (Mettler Toledo AG, Zürich, Switzerland) coupled with a Huber TC100 cooler (Offenburg, Germany) was applied to examine the amorphous character of the electrospun sample. The DSC measurements were performed in a stochastic temperature-modulated mode called TOPEM^®^, which enabled the separation of the total heat flow to reversing- and non-reversing components. During the TOPEM^®^ measuring process, the frequency of the modulation varies randomly within a given range, in this case, between 15 and 30 s. The temperature was raised from 0 °C to 250 °C while the overall heating rate was 2 °C/min. The pulse height was set to 1 °C, meaning that the temperature was modulated continuously by ±0.5 °C. The DSC chamber was purged with dry nitrogen using a flow rate of 50 mL/h. For the DSC experiments, a sample weight of 7–15 mg was measured into a 40 µL pierced aluminum pan.

To determine the T_g_ of the amorphous NAP, the traditional linear heating DSC method was applied from −50 °C to 250 °C with a heating rate of 20 °C/min. The nitrogen flush, the sample mass, and the pan were the same as used during the modulated measurements.

### 2.6. Thermogravimetric Analysis (TGA)

The residual solvent content of the samples was investigated by thermogravimetric analysis using a Q5000 TGA instrument (TA Instruments, New Castle, DE, USA). Ca. 10 mg electrospun sample was measured into a tared platinum pan. The adjusted heating program increased the temperature of the chamber from 20 to 105 °C at 2 °C/min, which was followed by an isothermal part at 105 °C for a further 15 min. Nitrogen flush with a rate of 25 mL/min was introduced into the chamber during the measurements.

### 2.7. Thermally Stimulated Depolarization Current (TSDC)

**Figure 1:**
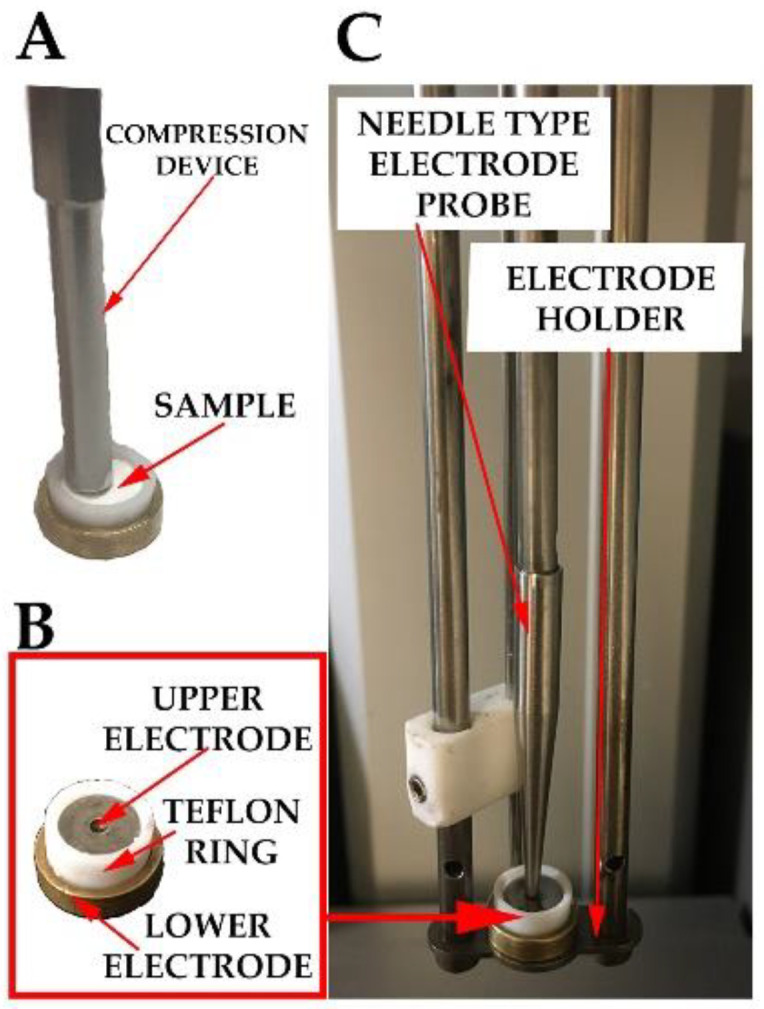
Photos of the TSCII setup ((**A**) measured and compressed sample; (**B**) measuring cell containing the sample; (**C**) measuring cell inserted into the TSCII device).

TSDC measurements were performed with a TSCII-type thermally stimulated current equipment (Setaram, Calure, France). The device used liquid nitrogen as a coolant, which was controlled by a Norhof 910 LN2 pump (Ede, The Nederlands). The measuring chamber was purged with helium three times before each measurement to ensure an inert atmosphere during the experiment. For the TSDC experiments, ca. 18–20 mg electrospun material was measured and compressed manually into a copper sample holder with a Teflon ring, which was applied as the bottom electrode (Figure 1A). Preliminary works showed that this amount corresponded to the 0.5 mm sample thickness. After the sample preparation, a stainless steel disc used as an upper electrode was placed on the surface of the sample (Figure 1B). The closed sample holder was put in the equipment between a needle-type electrode probe and an electrode holder (Figure 1C). The effective area of the prepared parallel plane capacitor was 38.5 mm^2^ (meaning that the diameter of the upper electrode was 7 mm).

**Figure 2:**
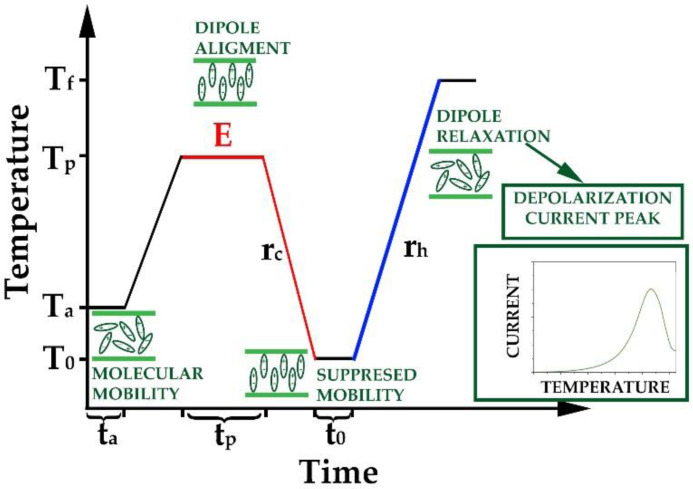
Schematic drawing of the applied measuring process. (Red lines indicate the part of the measurement where field strength (marked with red E) was turned on. The blue line indicates the depolarization step when the depolarization current was recorded. The green partial figures show the arrangement of the dipoles during the different processing steps.

Figure 2 presents the scheme of the applied measuring process, during which the depolarization current induced by molecular mobility was determined [37]. During all measurements, the annealing temperature (T_a_) was adjusted to 20 °C, and the annealing time (t_a_) was 1 min. After holding the sample at the annealing temperature, the measuring program continued with a heating step to reach the polarization temperature (T_p_). T_p_ for each composition was set such that it was ca. 20 °C higher than its T_g_ determined by DSC [37]. The polarization time (t_p_) was 5 min in all cases, while 300 V/mm field strength (E) was applied, which meant 150 V polarization voltage (U_p_) for the 0.5 mm sample thickness. The polarization was followed by a cooling step with a 10 °C/min cooling rate (r_c_) to the 0 °C (T_0_). The electric field was still present during the cooling step. The holding time at T_0_ (t_0_) was set to 1 min while the polarization field was removed. Then a heating step was followed with a heating rate of 5 °C/min (r_h_) to reach the final temperature (T_f_ = 150 °C). The depolarization current was measured during the heating and was depicted as a function of the temperature.

### 2.8. Micro-Thermal Analysis (µTA)

The third method to determine the T_g_ of the samples was µTA™. The measurements were accomplished using a 2990 Micro-Thermal Analyzer (TA Instruments, New Castle, DE, USA) [46] with µTALab software (SPM Labs Llc., Tempe, AZ, USA). The key element of the device is the atomic force microscopic (AFM) head with an extremely tiny temperature probe. Since the prepared electrospun fibers had a very loose and soft structure, stabilizing the probe on the surface of the sample before the measurement was not possible. Therefore, fibers were compressed first using the measuring cell of the TSDC. Later, flat round pastilles were formed with a Camilla OL95-type press by using 100 bar pressure. Approximately 50–55 mg samples were used for this to reach ~0.5 mm sample thickness. Then ca. 1 × 1 mm pieces were cut from the pastilles and stuck with double-sided adhesive tape on the surface of a 1 cm^2^ metal disk, which was placed under the thermal probe. The surface of the electrospun sample was investigated by a microscope coupled with a charged coupled device (CCD) camera. This accessory facilitated the selection of the measuring points. The temperature probe was heated from 0 °C to 180 °C with a heating rate of 10 °C/s. Five points were measured on each investigated surface, and two different pieces were investigated from each composition.

### 2.9. Stress Stability Test

A short-term stress stability test was accomplished with the different ASDs to examine their behavior after storage at a higher temperature, and higher relative humidity (RH). The samples were placed into a Pol-Eko KK115 type climate chamber (Pol-Eko Apparatura, Poland) at 30 °C and 65% RH in open holders for 1 week. Sampling was done after 1 day and 1 week. The stability samples were investigated by SEM, DSC, TSDC, µTA, and XRPD.

### 2.10. Polarized Light Microscopy (PLM)

The presence of crystalline traces in the samples after the stress stability test was analyzed by an Amplival Carl Zeiss (Jena, Germany) polarized microscope equipped with an OLYMPUS C4040 Z-type camera. A DP-Soft software was applied for recording PLM images and evaluation. The agglomerated electrospun materials were distributed in silicon oil before the measurements.

### 2.11. In Vitro Dissolution Testing

The dissolution test of the different electrospun samples was carried out with a Pharmatest PTWS 600 dissolution tester (Pharma Test Apparatebau AG, Hainburg, Germany). The samples were examined using the so-called “tapped basket” method, which means the combination of the USP I and the USP II dissolution tests [47]. The adjusted stirrer speed of the paddles was 50 rpm. For the dissolution tests, 900 mL of 0.1 N HCl dissolution media at 37 ± 0.5 °C was used. The investigated dose was 50 mg, while each composition was measured in triplicate. Online measurement of the dissolved NAP was performed using an Agilent 8453 UV–Vis spectrophotometer (Agilent Technologies, Santa Clara, CA, USA) at 272 nm and a flow cell system equipped with a length of 10 mm cuvettes.

## 3. Results and Discussion

### 3.1. Preparation and Basic Characterization of the Samples

The preparation of appropriate ASDs is the key condition to evaluate the contribution of different thermoanalytical methods to the characterization of the glass transition process. Electrospinning, being able to form a perfect amorphous structure, was chosen for the sample preparation in this research. This ASD preparation method requires less solvent than other methods, the electrostatic field facilitates, the evaporation of the solvents, and the electrospinning can be performed at room temperature and atmospheric pressure; therefore, it is a very gentle way of the preparation of amorphous pharmaceutical solids. Table 1 summarizes the details of the different ASD’s examined in this work.

**Figure 3:**
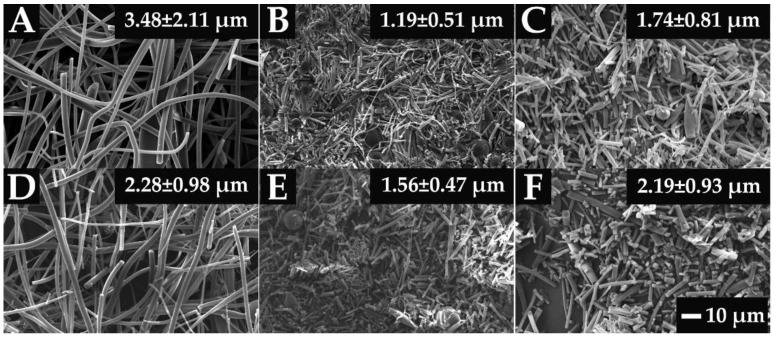
SEM images of the prepared electrospun samples at a magnification of 1000×. The average fiber diameters are indicated on the images ((**A**) PVPVA64, (**B**) 10%NAP_90%PVPVA64, (**C**) 20%NAP_80%PVPVA64, (**D**) 30%NAP_70%PVPVA64, (**E**) 40%NAP_60%PVPVA64, (**F**) 50%NAP_50%PVPVA64).

Another advantage of electrospinning is that changes in the morphology of the fibers might be visible indicators of the instability of the ASD systems [48]. Considering the SEM images of the different ASDs after the electrospinning (Figure 3), it can be seen that the fiber formation was successfully achieved in all cases. Although the fiber diameter of the NAP-loaded samples is slightly smaller than the PVPVA64 fibers, the diameter of each composition falls in a similar size range. The thinner fibers observed in the case of the API- containing samples suggest that NAP probably increased the conductivity of the solution, thus helping the elongation of the jets during the electrospinning process [49].

**Figure 4:**
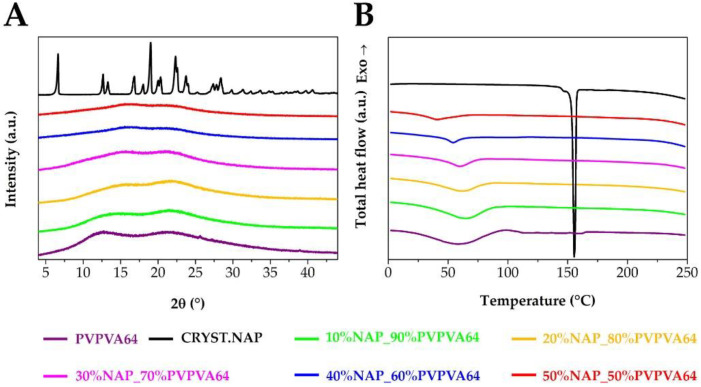
The (**A**,**B**) parts of the Figure present XRPD patterns and DSC thermograms of the samples with different compositions and references, respectively.

However, the main question that arises is whether the formation of the amorphous arrangement was successful. The XRPD patterns of the fibrous samples showed only the amorphous halo and no characteristic Bragg peaks of the crystalline NAP (Figure 4A), which suggested that the amorphization was successfully fulfilled via electrospinning. This was confirmed by the DSC measurements as no melting peak of the API was observed in the thermograms of the electrospun samples (Figure 4B). Although flatter peaks were seen at lower temperatures, those belonged to other events such as the enthalpy relaxation, the presence of moisture in the samples, and the glass transitions. As the polymer content decreased, the size of the integrated peak also decreased.

**Figure 5:**
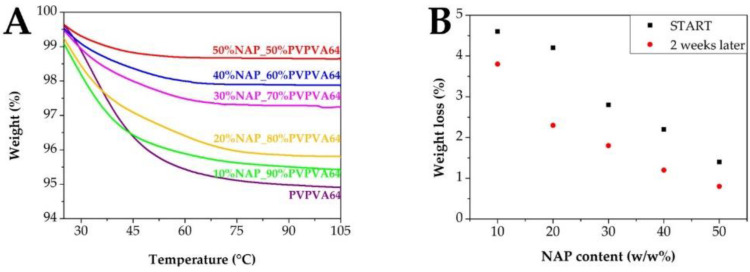
Investigation of the moisture content of the samples by TGA. (**A**) shows the changes in weight right after the electrospinning while (**B**) compares the weight losses of samples right after the electrospinning and after 2 weeks of drying at room temperature.

The effect of the moisture content was investigated by TGA as well (Figure 5), which correlated well with the DSC results. The smallest peak area belonged to the lowest polymer content sample in the case of the DSC thermograms, while that composition was characterized by the lowest weight loss by the TGA measurements. This suggests that the moisture content of the samples can be linked to the polymer, which can influence the glass transition temperature and also the physical stability of the ASDs [50]. To exclude the effect of the residual solvents coming from the solvent-based electrospinning, the samples were investigated 2 weeks later of the preparation process as well (Figure 5B). Although the weight loss of the samples decreased after drying at room temperature, suggesting the evaporation of the residual solvents, the decreasing tendency in the function of the NAP content remained.

Summarizing the results of the basic characterizations it can be said that the preparation of ASDs was successfully performed, and the samples proved to be suitable for further investigations.

### 3.2. Measuring the Glass Transition Temperature with Different Methods

**Figure 6:**
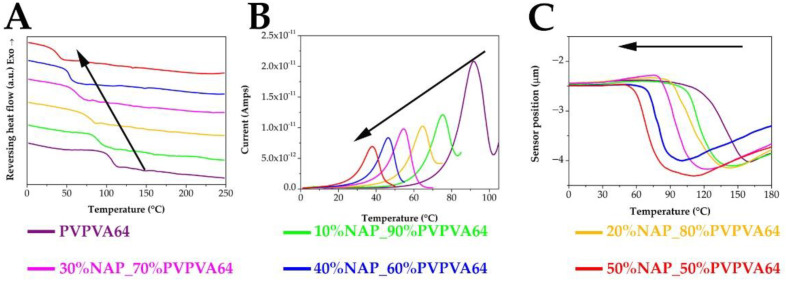
Summary of the MDSC (**A**), TSDC (**B**), and µTA (**C**) results for determining T_g_. (The black arrows indicate the direction of the decreasing tendency in the T_g_ values).

The main goal of this research was to evaluate three thermoanalytical methods, working on different principles, for characterizing amorphous pharmaceuticals. Therefore, the next step was to investigate the glass transition of the prepared electrospun samples and determine the T_g_ values based on the measurements. Figure 6 proves that each technique was suitable to indicate clearly one signal related to the glass transition. As it was mentioned above, MDSC makes it possible to separate the overlapping events and present the glass transition in the reversible heat flow. Taking advantage of this, T_g_ values of the NAP-loaded samples were easily determined from the ramp belonging to the glass transition of the ASDs (Figure 6A). Similarly, µTA indicates the glass transition with a ramp too, because the sensor position decreases with increased molecular mobility and free volume (Figure 6C). µTA was able to measure more points of the sample in a few seconds, enabling the examination of the micro homogeneity of the samples. The relative standard deviations were between one and six percent, indicating the homogeneous distribution of the API in the polymer matrix. During the TSDC measurements, a well-visible peak corresponds to the T_g_ since the depolarization current starts to increase when the ordered dipoles start to rearrange to the disordered state (Figure 6B). As all techniques showed only one single ramp (DSC, µTA) or peak (TSDC), the results confirmed that the amorphization was successfully achieved. The absence of phase separation indicated that the NAP is molecularly dispersed in the PVPVA64. Looking at the T_g_ results of the three different measurements together, it is clear that the same decreasing tendency can be observed in the case of each method as the API content is increasing. This trend refers to the plasticizing effect of the NAP, which was efficiently detectable with all the tested analytical techniques.

**Figure 7:**
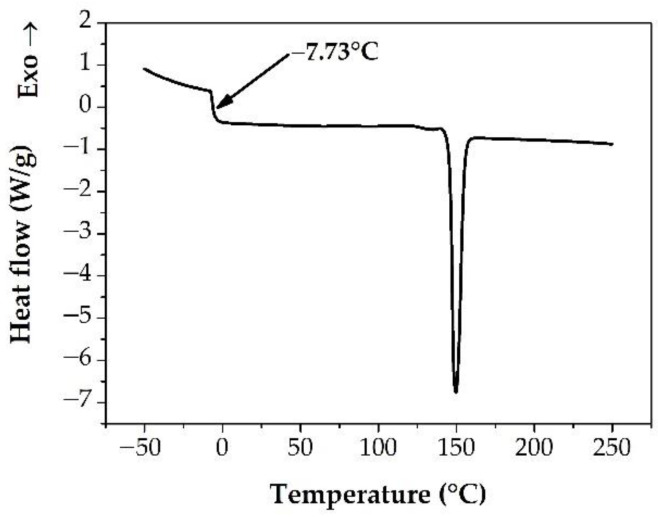
DSC thermogram of the measurement for T_g_ determination of amorphous NAP.

However, the T_g_ of the pure API is missing from Figure 6 because that could not be determined by the applied methods. Two different amorphization techniques were tested to prepare the amorphous NAP. First, a quench cooling process was tried. The crystalline API was heated to 30 °C above its melting point, and then it was cooled with liquid nitrogen. The prepared sample was then measured into a DSC pan, and the same MDSC method was adjusted as used in the case of the ASDs. Not surprisingly, the ambient temperature while the samples were measured and the slow heating rate during the MDSC measurement was favorable to the crystallization. Consequently, only the melting peak of the crystalline API was observed in the thermograms (data are not shown). For this reason, amorphous NAP was attempted to form in-situ in the DSC chamber. To achieve this, the crystalline API measured into the DSC pan was heated to 30 °C above its melting point, and then it was cooled down with the maximum cooling rate of the device (~40 °C/min) to −70 °C. The MDSC method resulted in only the melting peak of the NAP again, indicating that the rate of temperature change, applicable with this method, is slow to detect the glass transition in the case of this prone to crystallization of the API (data are not shown). In order to avoid crystallization, finally, the in-situ prepared amorphous NAP was investigated with the conventional DSC method and faster heating rate (20 °C/min). This measurement method proved to be suitable for detecting the glass transition of the amorphous NAP (Figure 7). Although the sample was not fully amorphous, as the melting peak was also visible in the thermogram, a clear ramp appeared under 0 °C, indicating the T_g_ of the amorphous part of the investigated NAP. The determined T_g_ value is the same as the estimated result described in the [51] publication confirming the theoretical calculations experimentally. However, it is worth considering that the amorphization (applied method, cooling rate, etc.) can affect the measured T_g_ value; therefore, the T_g_ measured in this study might differ from other already published values [52,53]. Despite this, regarding the determination of the T_g_ of the pure NAP, DSC proved to be the most suitable technique among the three tested methods. The in-situ sample preparation could not be performed in the case of the µTA device, while (although the in-situ sample preparation was possible) in the TSDC equipment the huge changes in the free volume resulted in the loss of contact between the sample and the electrode, generating a noisy and uninterpretable curve (data are not shown).

On the whole, however, T_g_ of all ASDs could be measured by each investigated thermoanalytical technique. In addition, the determination of the T_g_ of the pure NAP enabled to use of the Gordon-Taylor equation (Equation (1)) for calculating the theoretical T_g_ values of the ASD samples [51,54].

T_g,mix_ = (w_d_T_g,d_ + Kw_p_T_g,p_)/(w_d_ + Kw_p_) (1)

In Equation (1), w_d_ and w_p_ indicate the weight fraction of the drug and the polymer, respectively. T_g,mix_, T_g,d_, and T_g,p_ refer to the T_g_ of the ASD, pure drug, and the polymer, respectively. Furthermore, K is a constant, which can be determined using the density of amorphous drug (ρ_d_) and polymer (ρ_p_) based on Equation (2).

K ≈ (ρ_d_T_g,d_/ρ_p_T_g,p_) (2)

**Figure 8:**
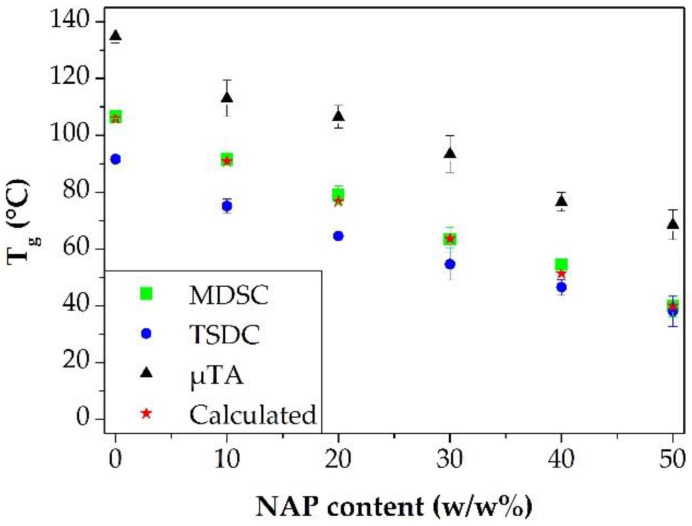
Comparison of the T_g_ values measured by different techniques and investigation of their correlation with the calculated theoretical values determined by the Gordon-Taylor equation.

Using the density data from the publication [51], the theoretical T_g_ of the prepared ASDs was determined according to the Gordon-Taylor equation and utilizing the measured T_g_ of the pure NAP and PVPVA64 by DSC. The calculated and the measured T_g_ values were compared in Figure 8. Based on this comparison, it can be concluded that the DSC results are most close to the calculated T_g_ values. This may not be surprising considering that the T_g_ values of the pure components used for the calculation were determined by DSC, it is logical. However, why the values measured by the other two methods differ so much and in such directions is questionable.

It is considered to be obvious that there are some differences between the results of the three different techniques because their measuring principles are distinct. TSDC analysis uses an electric field that can excite other parts of the molecule too in contrast to the simple increase in temperature used by the other two methods [35]. Furthermore, the effect of mechanical loading in the case of µTA can also influence the locality of the T_g_ [55]. During the TSDC measurements, the plan is to approach the heating rate of the MDSC. However, heating that is too slow is not recommended because the peaks relating to phase separation can disappear [56]. That is why 5 °C/min was adjusted as the heating rate during the TSDC measurements. Despite the higher heating rate, lower temperatures were measured than in the case of the DSC measurements, especially at lower API content. It is well known that the Gordon-Taylor equation does not give a precise prediction to T_g_ if intermolecular bonds occur in the system [57], for instance, in the case of ASDs containing NAP and PVPVA64. Since the T_g_ values measured by DSC are very close to the calculated values (less than 5 °C differences were by each composition) it can be assumed that the DSC did not detect or hardly detected the interaction between the API and the polymer (Table 2). In contrast, the values measured by the TSDC method reflect the interactions between the NAP and the PVPVA64, suggesting that the presence of intermolecular interactions can be better detected with this technique.

**Figure 9:**
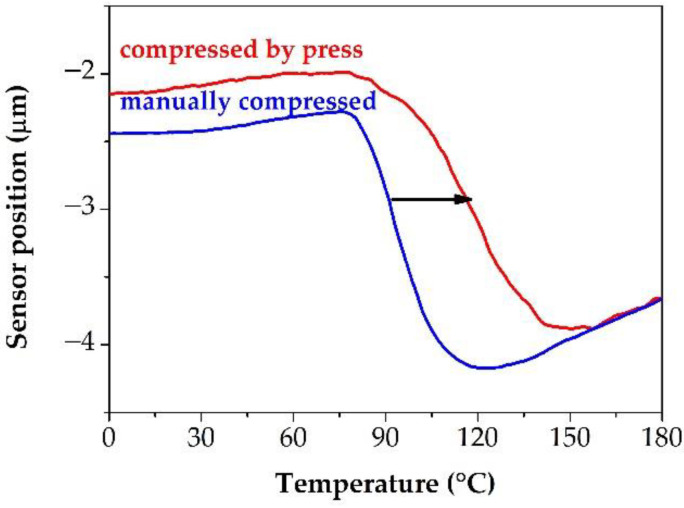
Exploration of the reason for the different T_g_ values measured by µTA. (The results of the 30%NAP–70%PVPVA64 sample are presented here to demonstrate the effects in question. The black arrow indicates the direction of change due to higher pressure).

In contrast to TSDC, µTA showed higher values than the DSC measurements. One of the reasons can be the measuring principle; instead of the whole sample only very small volumes (touched by the thermal probe) are heated during the µTA measurement. On the other hand, compression applied on the measured fibers could contribute to the higher T_g_ values. The effect of the compression was investigated in-depth. It was observed that the compression significantly affects the measured glass transition (Figure 9); with increasing compression force, the determined T_g_ increased as well. Although in this way, the differences in the T_g_ values measured by DSC and µTA are increasing too, the samples compressed by the press were used during the further experiments because this sample preparation is more reproducible. Otherwise, it could be challenging to distinguish the differences that come from the distinct composition and from the compression, which could led to false results. In addition, the tendency was similar to that shown in Figure 6C (see later in Section 3.3). Consequently, samples compressed at 100 bar proved to be suitable for further experiments.

Taking into account the results of the three different techniques to measure T_g_, it can be stated that all of them are suitable for determining the T_g_ of API-loaded ASDs. Nevertheless, each method has an advantage compared to the other two. The DSC proved to be the only applicable method for measuring the T_g_ of the amorphous NAP. TSDC results were influenced by intermolecular interactions. Finally, µTA provided the fastest results, making it feasible to measure repetitions in a very short time, determining the homogeneity of the samples and indicating the effect of mechanical stresses.

### 3.3. Effect of the Stress Stability Tests

It was clearly visible that all tested analytical techniques are appropriate for investigating the ASDs containing NAP and PVPVA64. To see their relevance in the examination of the ASD systems’ physical stability, the prepared samples were stored at stress stability conditions. Overviewing the SEM images, it is already conspicuous that the plasticizer effect of the API does not favor the morphology of the fibers (Table 3). While the electrospun polymer kept its fibrous structure even after one week of storage, the ASD samples lost it. With increasing API content, the merging of the fibers and the disappearance of the fibrous structure happened earlier. The samples with 10% and 20% drug loading remained fibrous after one day of storage. The diameter of the fibers started to increase, and fibers were united in the case of the sample containing 30% of NAP after the first day. The samples with the highest API loading lost their fibrous characteristics right after one day of storage. Considering the SEM images, it can be expected that the T_g_ values may be changed since the samples reacted spectacularly to the increased moisture content in their environment [58].

**Figure 10:**
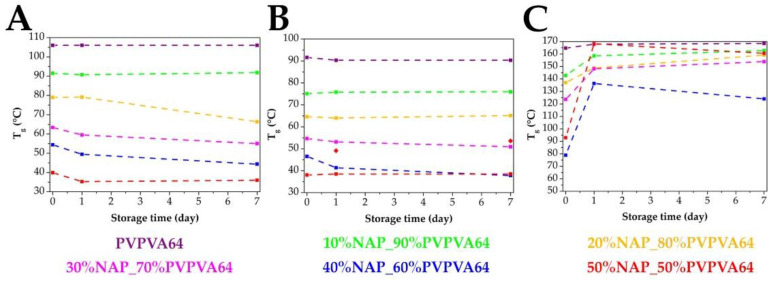
Changes of the T_g_ values measured by MDSC (**A**), TSDC (**B**), and µTA (**C**) in the function of the storage time.

Contrary to expectations, the T_g_ values did not change in all cases after the stress stability tests (Figure 10). According to the MDSC measurements, only those with an API content of 20% or more showed a decreasing tendency in the T_g_ values; no significant changes were detectable during the measurements of 10% NAP-loaded samples (Figure 10A). Similar trends were seen in the case of the TSDC results, with the difference that only the results of the samples with drug content of 30% and more showed a decrease in the T_g_ values after the storage (Figure 10B). Another interesting fact about the TSDC results is that in the case of the 50% drug loading sample, in the T_g_ region measured before storage, only an inflection of a curve can be observed after the stress stability test, but one clear peak appeared at a higher temperature. That is why more red points can be seen in Figure 10B, which can be explained as the signal of phase separation in the highest API-loaded sample. The most significant shifts were measured by µTA (Figure 10C). In contrast with the other two methods, higher T_g_ values were experienced with µTA after the stress stability tests. Only the T_g_ of the polymer did not change, which is well correlated to the SEM images and the results of the other two methods. Looking at the results of the ASDs, the T_g_-s increased in the direction of the T_g_ of the PVPVA64, which assumes that the phase separation started [39]. One of the reasons that higher drug-loading samples stand out more might be that these ASDs started to crystallize, which can distort the evaluation. In addition, it is also visible that the order of the T_g_ before the storage in the case of the 40% and 50% drug-containing samples were exchanged compared to the compressed samples. This difference indicates that the compression force had an influence on the stability of the samples [59].

**Figure 11:**
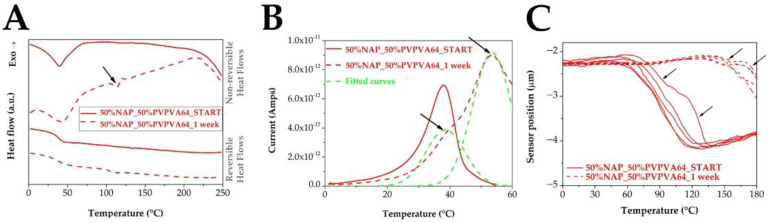
In-depth investigation of the DSC thermograms (**A**), the depolarization current peaks (**B**), and the ramps measured by µTA (**C**) of the 50% API containing ASDs before and after 1 week of storage. (Black arrows indicate signs of phase separation and crystallization).

However, despite the changes in the T_g_ values, only the results of the 50% drug loading samples showed an extra signal pointing to phase separation (Figure 11). In the non-reversing heat flow of the MDSC thermogram, a peak was observed around 114 °C, which was followed by a small exothermic peak belonging to a cold crystallization (Figure 11A). A step change around 114 °C is also visible in the reversing heat flow of the sample after the stress stability tests indicating a glass transition, referring to the phase separation in the ASD. The TSDC results showed the above-mentioned inflection in the curve and the second peak, which are related to the phase separation as well (Figure 11B). Similarly, more than one ramp could see in the case of the µTA results too, representing that the molecular dispersity of the API in the polymer is not homogeneous anymore (Figure 11C). Indeed, the effect of the compression on the stability of the high NAP-loaded ASD was also visible since an additional ramp appeared even during the measurement of the samples before the storage. The investigation of the other compositions did not result in similar changes. Consequently, only the differences in the T_g_ values can be applied to predict the stability of the prepared ASD samples.

### 3.4. Correlations between the Measured T_g_ Values and the Stability

In the previous chapter, the changes in morphology and the T_g_ values after the stability test were highlighted. However, the investigation of crystalline traces, which could be evidence of physical instability, was not presented above. Therefore, in this section, the stability described by the absence or presence of crystalline material will be discussed. Furthermore, it will also be examined whether there is a correlation between the T_g_ values and the physical stability.

**Figure 12:**
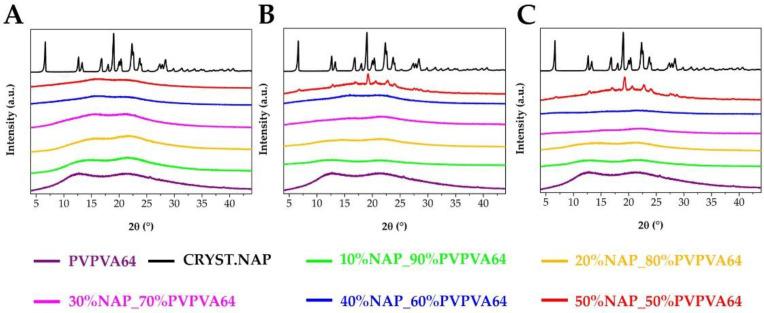
XRPD patterns of the ASD samples before storage (**A**), after 1 day storage (**B**), and after 1 week storage (**C**).

First, one of the most common methods, the XRPD was applied to investigate the NAP-loaded ASD samples before and after the storage (Figure 12). In connection with what was previously mentioned, the largest changes were observable during the measurement of the 50% drug loading sample. XRPD patterns of the samples after one day and one week of storage showed small Bragg peaks indicating the presence of crystallinity. All the other samples did not show Bragg peaks, which would mean two things. If the samples after the stress stability test contained crystalline traces, their amount is below the detection limit of the applied method at the adjusted parameters. The other possibility could be that the samples, except those with 50% drug loading, were stable after storage at stress conditions.

To investigate both possibilities, PLM was used. These measurements resulted in a more clear view (Table 4). Birefringence was seen in the samples containing 40% and 50% NAP after one day, while all of the prepared ASDs stored for one week at stress conditions showed crystalline traces. Consequently, the decreasing T_g_ values predict the instabilities of the samples as the higher NAP loading ASDs with the lowest T_g_-s can be characterized with the lowest physical stability. However, according to the T_g_ values measured after the storage at stress conditions, the physical instability of the sample containing 10% of NAP can be predictable only based on the µTA. The other two methods did not show any changes in the T_g_ of this sample.

**Figure 13:**
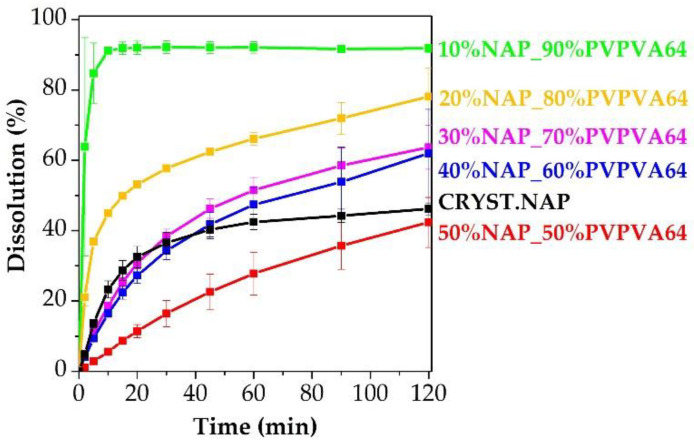
Dissolution results of the prepared ASDs. Applied parameters: 37 ± 0.5 °C, 900 mL of 0.1 M HCl dissolution medium, 50 mg of the API content, “tapped basket” method, 50 rpm, *n* = 3.

Finally, the other crucial factor, the dissolution, needed to be investigated since it can provide whether the prepared ASD enhanced the dissolution of the poorly water-soluble API. The dissolution results showed a good correlation with the measured T_g_ values (Figure 13). The ASD that showed the highest T_g_, reached the highest dissolution. In addition, a similar decreasing trend was observed in the dissolution percentages as in the case of the T_g_ values. These results can be in context with the drug-polymer ratio [60].

## 4. Conclusions

The present study demonstrated the special applicability of three different thermoanalytical techniques in the context of T_g_ determination and physical stability-related questions. The investigation of the T_g_ of the different ASDs highlighted that similar tendencies could be observed with MDSC, TSDC, and µTA. However, each method has some advantages compared to the other two techniques. The MDSC enabled distinguishing between the glass transition from the non-reversing events of the NAP-PVPVA64 ASDs’. Indeed, the T_g_ of the pure NAP was also successfully determined by DSC. TSDC proved to be suitable to highlight the interactions between the polymer and the API since the deviations from the theoretical values calculated by the Gordon-Taylor equation were higher compared to the T_g_ values measured by MDSC. Finally, the main advantage of the µTA lies in its speed, which contributes to the investigation of the micro homogeneity of the ASDs via measuring more points within a few seconds. The relative standard deviation of the repeated measurements was less than 6% in all cases, indicating that all prepared samples were homogeneous right after the electrospinning.

After the stress stability test, it was clear that the three thermoanalytical methods showed the changes in the T_g_ values in a different way. According to the MDSC results, the T_g_ decreased in the function of storage time in the case of the samples containing 20% or more API. The TSDC was able to detect this decreasing tendency only from the 30% and more drug-loading samples. In contrast with the MDSC and TSDC, however, µTA indicated changes even in the T_g_ values of the sample containing 10% NAP. Considering the PLM images, where the ASD containing 10% API also showed crystalline traces after one week of storage, T_g_ values measured by µTA were the most suggestive of the samples’ instability.

The similarities and the additional opportunities drew attention to the fact that it may be worthwhile to use these methods simultaneously. The joint application of more analytical techniques can help to understand the behavior of the ASD samples, which can be really challenging, especially in the early stage of development. Furthermore, the results revealed that stability might be studied with minimal material consumption and fast analytical methods. Indeed, the dissolution percentages showed a similar decreasing trend to the measured T_g,_ indicating that the API content has a key role in the case of ASDs containing NAP and PVPVA64. Overviewing the outputs of this research, it can be concluded that better knowledge of ASD systems can be achieved through the information of suitable analytical methods. In conclusion, accelerated developments and high-quality ASD-loaded medicine production could be achieved thanks to the in-depth understanding of the behavior of ASDs.

## Figures and Tables

**Table 1:** Summary of the prepared NAP-PVPVA64 compositions.

Sample Code	NAP (mg)	PVPVA64 (mg)	Ratio of the Solvents (*v*/*v*)	Amount of the Solvents
PVPVA64	-	2000.0	DCM:EtOH 1:1	8 mL
10%NAP_90%PVPVA64	222.2			
20%NAP_80%PVPVA64	500.0			
30%NAP_70%PVPVA64	857.1			
40%NAP_60%PVPVA64	1333.3			
50%NAP_50%PVPVA64	2000.0			
CRYST. NAP	Crystalline NAP was used as a reference as received, without any modification.			

**Table 2:** Deviation of the measured T_g_ values from the calculated ones.

Sample	MDSC (°C)	TSDC (°C)
PVPVA64	0	14
10%NAP_90%PVPVA64	0	16
20%NAP_80%PVPVA64	−5	12
30%NAP_70%PVPVA64	−1	9
40%NAP_60%PVPVA64	−2	5
50%NAP_50%PVPVA64	1	2

**Table 3:** Summary of the SEM images of the prepared samples before and after storage.

	START	1 Day	1 Week
PVPVA64	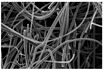	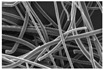	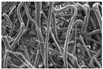
10%NAP_ 90%PVPVA64	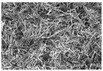	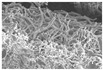	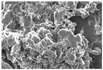
20%NAP_ 80%PVPVA64	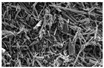	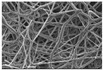	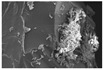
30%NAP_ 70%PVPVA64	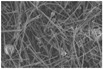	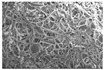	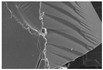
40%NAP_ 60%PVPVA64	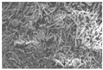	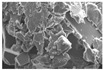	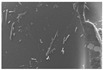
50%NAP_ 50%PVPVA64	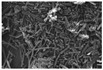	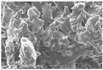	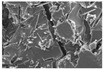

**Table 4:** PLM images of the samples before and after the stress stability test, and the images of the reference materials.

	START	1 Day	1 Week
10%NAP_ 90%PVPVA64	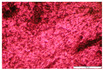	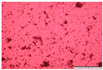	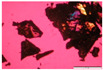
20%NAP_ 80%PVPVA64	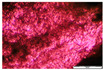	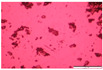	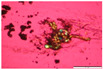
30%NAP_ 70%PVPVA64	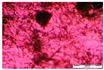	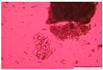	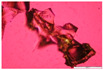
40%NAP_ 60%PVPVA64	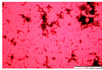	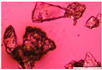	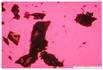
50%NAP_ 50%PVPVA64	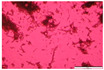	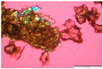	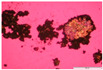
CRYST. NAP	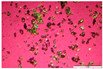	PVPVA64	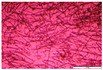

## Data Availability

Not applicable.
